# Comparison of Efficacy among Three Dermal Substitutes in the Management of Critical Lower-Limb Wounds: The Largest Biases-Reduced Single-Center Retrospective Cohort Study in Literature

**DOI:** 10.3390/medicina57121367

**Published:** 2021-12-15

**Authors:** Giuseppe Cottone, Francesco Amendola, Carlo Strada, Maria Chiara Bagnato, Roberto Brambilla, Francesco De Francesco, Luca Vaienti

**Affiliations:** 1 I.R.C.C.S. Istituto Ortopedico Galeazzi, Via Riccardo Galeazzi 4, 20126 Milan, Italy; gcottone.md@gmail.com (G.C.); fgsamendola@gmail.com (F.A.); luca.vaienti@unimi.it (L.V.); 2 Department of Biomedical Sciences for Health, University of Milan, Via Festa del Perdono 7, 20122 Milan, Italy; carlostrada@hotmail.it (C.S.); bagnatomariachiara@yahoo.it (M.C.B.); 3 Istituti Clinici Zucchi, Via Bartolomeo Zucchi 24, 20052 Monza, Italy; roberto.brambilla@grupposandonato.it; 4 Hand Surgery Unit, Department of Plastic and Reconstructive Surgery, Azienda “Ospedali Riuniti”, Via Conca 21, 60126 Ancona, Italy

**Keywords:** dermal substitute, Integra, Pelnac, Nevelia, lower limb wounds

## Abstract

*Background and objectives*: The skin recently became the main focus of regenerative medicine and, in this context, skin substitutes are fully entering into the plastic surgeon’s armamentarium. Among the various types of skin substitutes, dermal substitutes (DSs) are the most used. Our study aims to retrospectively compare three renowned and extremely similar DS in the management of critical lower limb wounds in the largest cohort analysis currently present in literature. *Materials and Methods*: We followed a strict protocol of application and evaluation of the DS for each patient and wound and, after a meticulous bias reduction process, we compared final outcomes in terms of efficacy and speed in achieving the defect coverage. *Results*: Among patients who did not receive a skin graft after the DS, we registered a wound healed surface of 50% for Pelnac, 52% for Integra, and 19% for Nevelia, after 30 days from the external silicon layer removal; among those who received a skin graft after the DS, we observed a significantly lower mean percentage of graft take after 7 days with Pelnac (53%) compared to Integra and Nevelia (92% and 80%, respectively). The overall percentage of wound healed surface obtained after 30 days from the external silicon sheet removal, either with or without skin graft, was 71% for Pelnac, 63% for Integra and 63% for Nevelia. We also ran a sub-group analysis only including grafted wounds with a negative microbiological test and the mean percentage of graft take was similar this time. Eventually, we assessed the influence of the wound’s “chronicity” on its healing, comparing the mean graft take only in “acute” wounds who received a skin graft and it resulted 63% for Pelnac, 91% for Integra and 75% for Nevelia. *Conclusions*: Integra demonstrates the highest rate of skin graft viability and the highest rate of skin graft takes after 7 days. Pelnac shows the quickest induction of secondary healing in acute wounds. Nevelia is not different from Integra and shows a superior graft take compared to Pelnac, but features the lowest secondary healing induction rate. No differences exist between the three DSs in terms of wound healing after 30 days from the skin graft or from the removal of the external silicon layer.

## 1. Introduction

More than a century has passed since the famous German pathologist Rudolph Virchow described, for the first time, the skin as more than a simple membrane protecting our internal organs [1]. From that point on, over the years, the skin acquired an increasingly important role as well for clinicians as for surgeons. Recently it became particularly the main focus of regenerative medicine [2,3,4,5,6]. Plastic surgeons are learning to master regenerative techniques and products when facing patients affected by critical, challenging wounds.

We use the terms “critical” or “complex” to define a wound whose healing process is hindered by intrinsic or extrinsic factors (such as comorbid diseases, smoking, infections, wound etiology) or when it tends not to solve spontaneously. Even the wound depth plays an unequivocal role in the healing process, because extensive deep tissue loss leads to stem cell reservoir depletion.

A skilled and updated surgeon should approach these situations using the well-known “reconstructive ladder” approach and its modifications [7,8,9]. In this context, skin substitutes are gaining more and more popularity and are fully entering into the plastic surgeon’s armamentarium.

Among the various types of skin substitutes, dermal substitutes (DSs) are the most used and studied being relatively easy to produce in large quantities with low cost, easy to store, and easy to handle [10]. They are engineered collagen-based regenerative matrices placed in contact with the wound bed and enhancing the autologous and spontaneous regeneration of the human dermis [11]. Thanks to their silicon superficial layer, they also act as an epidermis-like coverage while the colonization of the dermal matrix takes place.

DSs discussed in this study have a deep layer constituted by a bovine origin (Integra^®^, Integra Life Sciences, Princeton, NJ, USA and Nevelia^®^, Symatese Aesthetics, Bornel, France) or porcine origin (Pelnac^®^, Gunze Corp., Osaka, Japan) 3-dimensional collagen matrix and a superficial layer made by a silicon sheet to control moisture loss from the wound and act as a temporary artificial epidermis. In particular, Integra^®^ consists of a regular matrix of bovine (cow) derived type-I collagen cross-linked fibers (with pores size ranging from 20 to 125 μm) and shark-derived chondroitin-6-sulphate glycosaminoglycan (GAG); Nevelia^®^ is a porous (20–125 μm) matrix of about 2 mm thickness made of stabilized native type-I collagen without added GAG and with a mechanically reinforced silicone sheet (with a polyester fabric); Pelnac^®^ is made of a porcine tendon-derived atelocollagen sponge layer, approximately 3 mm in thickness and an outer fenestrated as well as reinforced silicone film. In two-three weeks the collagen matrix completely integrates into the wound bed and the external layer is removed to perform the skin graft, finalizing the reconstructive process.

Several existing studies investigated DSs regenerative properties and compared different DSs in term of efficacy and aesthetic results both in vitro and in vivo [12,13,14]. Our study aims to retrospectively compare three renowned DSs (extremely similar both from a structural and functional point of view) in the management of critical lower limb wounds in the undoubtedly largest cohort analysis currently present in the scientific literature.

## 2. Materials and Methods

After the local ethical committee approval, we retrospectively reviewed records of all patients admitted to our department from January 2015 to May 2020, suffering from extremely challenging lower limb wounds that were repaired by means of a dermal substitute. Patients were not randomized for the type of treatment but they were “enrolled” consecutively. The same surgeon treated all patients. Our study aimed to retrospectively compare outcomes of three different renowned DSs (Pelnac^®^, Integra^®^ and Nevelia^®^) in terms of efficacy and speed in achieving the defect coverage. Inclusion criteria were: DS application for lower limbs wounds; existence of detailed photographic and historical records from the first visit to the last follow-up. Exclusion criteria were previously performed surgical interventions on the wound under consideration (such as debridement and/or prior skin graft or flap); previous application of other types of advanced wound care products (such as other DSs, dermal inductors or negative pressure devices); previously performed minimally invasive adjunctive therapies (such as lipofilling, platelet-rich plasma or hyaluronic acid injections); immunocompromised state or immunosuppressive drugs assumption.

Experts in the field anonymously and independently reviewed all the patients’ records. Included patients were merged after a first review, while the senior author carried out a second final review of the cases. Detailed pathological, pharmacological and surgical history was reported for each patient. Great attention was posed both to systemic comorbidities and local factors influencing the natural wounds healing process. Every wound was also classified as “acute” or “chronic”, based on its etiology and duration: we considered as “acute” all those wounds derived from a surgical excision or post-traumatic, which were covered with the DS within 7 days from their diagnosis; we considered as “chronic” all the remaining long-lasting wounds or ulcers from any cause.

Before the dermal matrix application, the injured surface was calculated by means of a software (SketchAndCalc©, FSC, Inc. Overland Park, KS, USA). Expert authors carried out the clinical evaluation of every wound.

According to our protocol, multiple tissue samples for microbiological examination were always collected before the surgical operation (in the consulting room), 10–14 days before the scheduled surgery: in case of positive results for pathogens, a targeted antimicrobial therapy was promptly initiated some days before the surgical intervention or at least on the day of surgery.

We also followed a strict protocol of application and evaluation (by means of the well-established “T.I.M.E.” protocol [15,16] to evaluate each wounds) of the DS for each patient and wound: initial surgical debridement (either for acute or chronic wounds) and application of the dermal matrix; removal of the external silicon layer after 21 days from the placing and simultaneous assessment for final skin graft feasibility; if patient-related or wound-related conditions did not allow a safe coverage with a skin graft, the wound was left to heal by secondary intention and the total wounded area was reassessed at 30 days from the removal of the external silicon layer; if a skin graft was feasible, it was performed by the senior author on the same day; the clinical assessment of the graft take was performed after 7 days and the wounded area was reassessed after 30 days, as well. The outcomes we took into considerations were the skin graft’s feasibility (i.e., a viable and reliable “neodermis”) rate after the silicon layer removal, the skin engraftment rate at 7 days and the wound healed surface at 30 days, both for skin-grafted and for secondarily healed wounds.

Statistical analysis was carried out by means of a computer software (IBM SPSS, Armonk, NY, USA): continuous variables were compared using the Student’s *t*-test and the ANOVA when normally distributed, and Mann–Whitney U test and Kruskal–Wallis test when not normally distributed. Categorical variables were compared with the Chi-Squared test. Alfa was set to 0.05.

## 3. Results

From January 2015 to May 2020 (65 months), we treated 453 patients affected by critical lower limb wounds by means of a dermal substitute. 331 patients were excluded from our study: 176 patients had incomplete recorded data, 97 patients received a prior different treatment, 23 were immunocompromised, 35 had injured surfaces impossible to analyze with our computer software. A total of 122 patients met all predetermined criteria and were included in the study.

Forty-eight males and 74 females were reviewed, with a median age of 79 years (IQR 67–84). Lesions involved the lower leg in 54 cases, the ankle in 41 cases, the Achilles tendon region in 10 cases, and the foot in 17 cases.

Pelnac^®^ Double Layer Artificial Dermis was used in 72 patients, Integra^®^ Dermal Regeneration Template was used in 28 patients and Nevelia^®^ Bi-Layer Matrix in 22 patients.

Every patient, on average, presented with three systemic comorbidities, with the most common being arterial hypertension (n. 72, 59%) and chronic venous insufficiency (n. 58, 48%). No significant differences were registered among patients who received different DSs in terms of age, sex, diabetes, peripheral vasculopathy, chronic venous insufficiency, nephropathy, liver disease, number of total comorbidities.

Soft tissue defects had an acute etiology in 58 cases (48%) and a chronic etiology in 64 cases (52%). The most common aetiologies were the surgical excision of a cutaneous lesion (n = 37, 30%) and the venous ulcer (n = 34, 28%). We registered a significantly higher proportion of Pelnac^®^ used for chronic wounds (n = 51, 71%) compared to Integra^®^ (n. 4, 14%) and Nevelia^®^ (n. 9, 41%; *p* < 0.001).

The depth of the wound was superficial (i.e., supra-fascial) in 101 (83%) cases and deep (i.e., sub-fascial, with muscle or periosteum exposure) in 21 (17%) cases. Pelnac^®^ (n = 67, 93%) and Integra^®^ (n = 21, 75%) were significantly more frequently used for superficial wounds compared to Nevelia^®^ (n = 13, 59%; *p* < 0.001). The average wounded surface was 15 cm^2^ (CI 12.5–17.5), with no significant differences between different DSs or etiologies groups. Details about the patients and DSs are outlined in Table 1.

A microbiological analysis was performed in 108 wounds, with 57 (53%) tested positive for pathogens. The most involved bacteria were Gram-positive Cocci (n. = 39). A significantly higher number of infected wounds received Pelnac^®^ (n = 46 out of 60, 77%) compared to Integra^®^ (n = 3 out of 27, 11%) and Nevelia^®^ (n = 8 out of 21, 38%; *p* < 0.001).

After 21 days from the DS application, only 57 wounds were judged eligible to receive a skin graft; among those, 17 (24%) wounds that were covered with Pelnac received a skin graft, while Integra^®^ (n = 25, 89%) and Nevelia^®^ (n = 15, 68%) had a significantly higher rate of skin grafts (*p* < 0.001). Pelnac^®^ resulted also in a significantly lower mean percentage of graft take after 7 days (53%) compared to Integra^®^ and Nevelia^®^ (92% and 80%, respectively; *p* = 0.01).

Among the 65 patients who did not receive a skin graft after the DS, we registered a wound healed surface of 50% for Pelnac^®^, 52% for Integra^®^, and 19% for Nevelia^®^, after 30 days from the external silicon layer removal. These differences were not statistically significant at the multivariate analysis but resulted significant when directly comparing Pelnac^®^ with Nevelia^®^ (*p* = 0.04, not assuming similar variances). Instead, when directly comparing Integra^®^ with Nevelia^®^ in terms of healing percentage of non-grafted wounds (19% vs. 52%), the difference was not significant and we believe this is because of the low number of non-grafted wounds case in the “Integra” group (n = 3).

**Figure 1:**
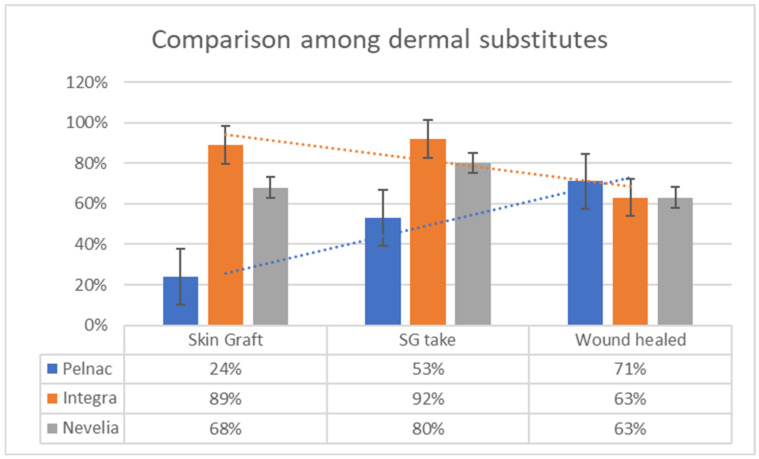
Comparison among dermal substitutes is summarized by means of bar charts. On the left is showed the skin graft feasibility evaluated after 21 days from the DS application (after the superficial silicone layer removal); in the center is showed the skin graft take percentage (in this case we obviously compared just skin-grafted wounds); on the right is showed the overall percentage of wound healed surface obtained after 30 days from the external silicon sheet removal (either with or without skin graft).

The overall percentage of wound healed surface obtained after 30 days from the external silicon sheet removal, either with or without skin graft, was 71% for Pelnac^®^, 63% for Integra and 63% for Nevelia^®^ (Figure 1).

We know that the etiology of the wounds (chronic vs. acute) and positivity to microbiological analysis could be possible confounding biases regarding the different rate of skin graft take and wound healed surface among different DSs groups. For this reason, we ran a sub-group analysis only including grafted wounds with a negative microbiological test (n = 7, 23, 11, respectively, for Pelnac^®^, Integra^®^, and Nevelia^®^); the mean percentage of graft take was similar this time: 57% for Pelnac^®^, 91% for Integra^®^, and 82% for Nevelia^®^; differences were not significant and we believe because of the inadequate sample dimension. We also compared the wound healed surface at 30 days in wounds with a negative microbiological result: 78% of wounds treated with Pelnac^®^ healed, 66% of those with Integra and 77% of those with Nevelia^®^, with a non-significant difference.

Only including superficial wounds (to reduce the “different depths”-related bias), the overall percentage of wound healed surfaces obtained after 30 days from the external silicon sheet removal was 94% for Pelnac^®^, 64% for Integra^®^ and 85% for Nevelia^®^ (these differences were statistically significant at the multivariate analysis, *p* = 0.00; they also resulted significant when directly comparing Pelnac^®^ with Integra^®^, *p* = 0.00, and Pelnac^®^ with Nevelia^®^, *p* = 0.03, while resulted non-significant when comparing Integra^®^ with Nevelia^®^, *p* = 0.30, not assuming similar variances).

Eventually, we assessed the influence of the wounds “chronicity” on its healing, comparing the graft take only in “acute” wounds who received a skin graft: a total of 8 acute wounds covered with Pelnac^®^, 22 with Integra^®^ and 12 with Nevelia^®^ received a skin graft; among those, the average skin graft take was 63% for Pelnac^®^, 91% for Integra^®^ and 75% for Nevelia^®^. It was not possible to compare the skin graft take among chronic wounds due to the low number of patients to run a reliable comparison.

**Figure 2:**
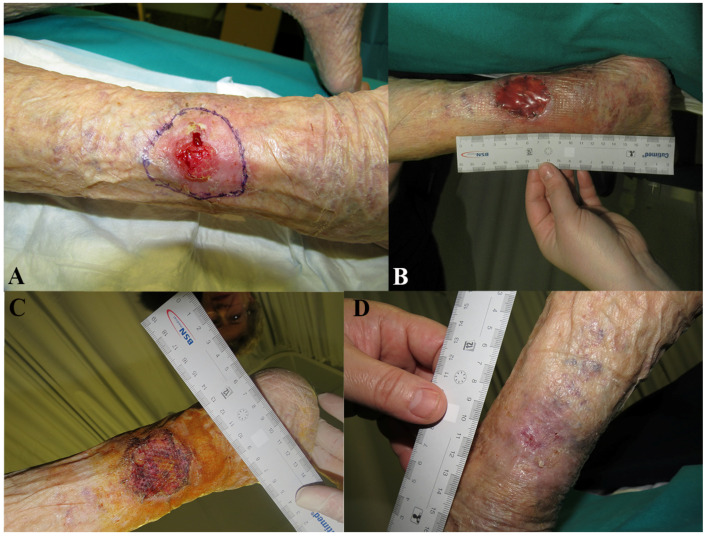
The figure shows a representative case of Integra^®^ dermal substitute, illustrating the initial wound appearance (**A**), the dermal substitute applying (**B**), the skin graft take (**C**) and the final closure result at 30 days (**D**).

**Figure 3:**
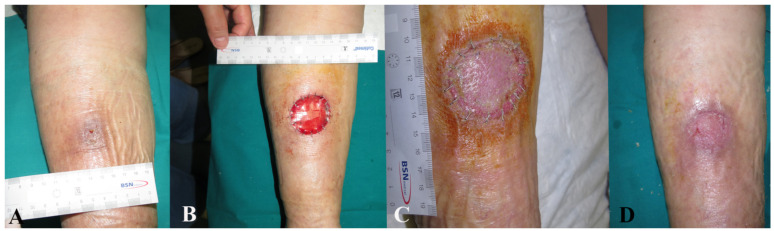
The figure shows a representative case of Integra^®^ dermal substitute, illustrating the initial wound appearance (**A**), the dermal substitute applying (**B**), the skin graft take (**C**) and the final closure result at 30 days (**D**).

**Figure 4:**
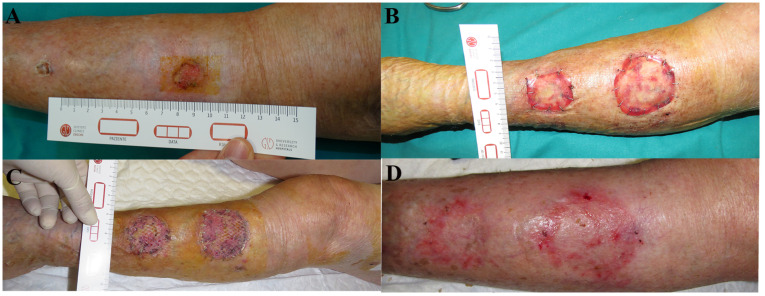
The figure shows a representative case of Nevelia^®^ dermal substitute, illustrating the initial wound appearance (**A**), the dermal substitute applying (**B**), the skin graft take (**C**) and the final closure result at 30 days (**D**).

**Figure 5:**
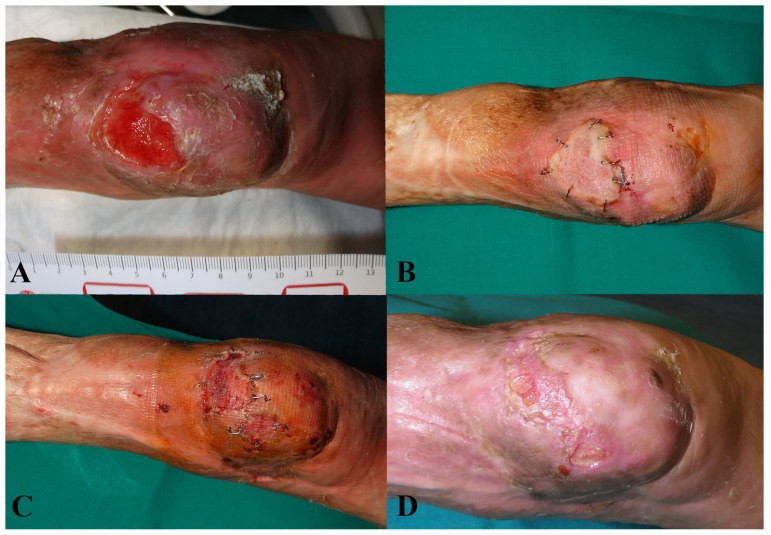
The figure shows a representative case of Nevelia^®^ dermal substitute, illustrating the initial wound appearance (**A**), the dermal substitute applying (**B**), the skin graft take (**C**) and the final closure result at 30 days (**D**).

**Figure 6:**
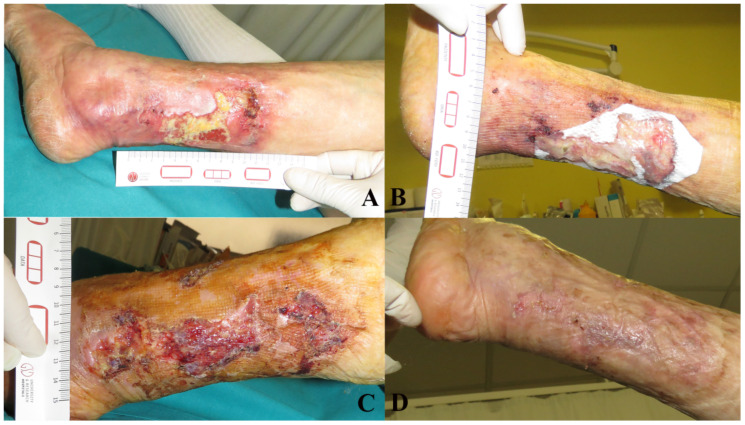
The figure shows a representative case of Pelnac^®^ dermal substitute, illustrating the initial wound appearance (**A**), the dermal substitute applying (**B**), the skin graft take (**C**) and the final closure result at 30 days (**D**).

**Figure 7:**
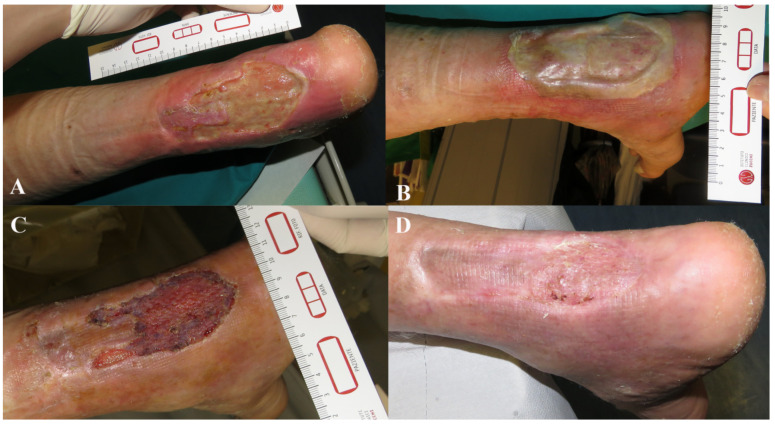
The figure shows a representative case of Pelnac^®^ dermal substitute, illustrating the initial wound appearance (**A**), the dermal substitute applying (**B**), the skin graft take (**C**) and the final closure result at 30 days (**D**).

It was not possible to attach all related photographic documentation, due to the great number of analyzed cases; for this reason we chose to show two representative cases for each dermal substitute (Figure 2 and Figure 3: Integra^®^; Figure 4 and Figure 5: Nevelia^®^; Figure 6 and Figure 7: Pelnac^®^) illustrating the initial wound appearance (A), the dermal substitute applying (B), the skin graft take (C) and the final closure result at 30 days (D).

## 4. Discussion

Dermal substitutes, boosted or not [14,17], represent an intermediate step in the reconstructive ladder, between a non-feasible skin graft and an avoidable flap. When applied on the wound “bed”, the dermal matrix is colonized by autologous fibroblast creating a viable scaffold onto which to perform a split-thickness skin graft. Their advantages are clear when deep structures, such as adipose tissue, muscles, tendons or bones, are exposed, even in the most challenging situations [18,19,20,21,22,23,24].

DSs showed in this study have a deep layer constituted by bovine origin (Integra^®^ and Nevelia^®^) or porcine origin (Pelnac^®^) 3-dimensional collagen matrix and a superficial layer made by a silicon sheet to control moisture loss from the wound and act as a temporary artificial epidermis. The three DSs showed differences in composition, structure, and storage adequacy. Integra^®^ is wet, sticky, and diaphanous. Pelnac^®^ is dehydrated, absorbent, and white, while Nevelia^®^ is porous and mechanically rigid. Integra^®^ possesses a thickness and pore size of 2 mm and 70–200 µm, Pelnac^®^ 3 mm and 70–110 µm, and Nevelia^®^ 2 mm and 200 µm.

Even if clear biochemical differences exist between different DSs [18], clinical superiority has not been established. Our retrospective large-cohort study aims to evaluate two different outcomes that, in our opinion, best reflect the definitive efficacy of DSs: the wound surface reduction (calculated after 30 days from the removal of the external layer) and the skin graft take (evaluated after seven days from the silicon sheet removal). This is the first multi-comparative in vivo study between different DSs and the undoubtedly largest present in literature.

The first outcome we considered is the percentage of wounds judged viable for a skin graft after the external silicon sheet removal: we are firmly convinced that this is an indirect and reliable measure of the “neodermis” quality. The “Pelnac” group received a skin graft in a significantly lower rate (24%) compared to “Integra” and “Nevelia” (89% and 68%, respectively); also, grafts on Pelnac^®^ resulted to take significantly less (53%) compared to Integra^®^ and Nevelia^®^ (92% and 80%, respectively).

Remarkably, when comparing the wound healed surface after 30 days from the external layer removal, either in the case of skin graft or not, Pelnac^®^ obtained a slightly higher rate of healing, although non-significant (71%, 63%, 63%, respectively) (Figure 1). This probably demonstrates that Pelnac^®^ induces a more rapid spontaneous re-epithelization of the wound, as already showed by De Francesco et al. [11], or a higher wounded surface contraction, as already demonstrated by Hori and colleagues [9]. After 30 days, wounds treated with Pelnac^®^ heal in the same way of Integra^®^ and Nevelia^®^, but starting from a lower rate of graft take.

One might state that Pelnac^®^ was more often used for chronic and infected wounds, compared to the other two substitutes, so to assess the influence of the infection [19], we compared DSs only in wounds with a negative microbiological test: even if not statistically significant, the mean percentage of graft take was similar to the one registered for undivided sample (57% for Pelnac^®^, 91% for Integra^®^, and 82% for Nevelia^®^). Our work showed that Pelnac^®^ has a greater bacterial-resistence compared to Integra^®^ and Nevelia^®^. This was just a scientific (as well as retrospective) observation, so we could just hypothesize this greater bacterial-resistence thinking to his outer reinforced and fenestrated silicone sheet that could resists against bacterial infection and meanwhile could facilitate the purulent exudation extrusion.

Another option could be the atelocollagen-based composition of this DS: the atelocollagen is a type-I collagen treated with proteases by cutting its extremities and in this way it results in a very pure compound rich in positive charges.

In addition, being well aware about the possible confounding bias related to the initial wound depth (as well as the small number of analyzed “deep” wounds), we also compared DSs only in “superficial” (i.e., supra-fascial) wounds and we surprisingly found a significant difference in term of percentage of overall wound healed surface obtained after 30 days from the external silicon sheet removal (94% for Pelnac^®^, 64% for Integra^®^ and 85% for Nevelia^®^).

Eventually, the comparison exclusively between skin-grafted “acute” wounds showed that Pelnac^®^ has a lower percentage of graft take (63%) but a higher rate of wound healing at 30 days (86%); even if not statistically significant, these percentages mirrored the same observed for the undivided sample and clearly showed a trend in a stronger secondary healing stimulation by Pelnac^®^. This trend is confirmed again by data regarding the healing of not-skin grafted wounds, in which both Pelnac^®^ and Integra^®^ demonstrated a higher percentage of closure compared to Nevelia^®^.

Being well aware about possible statistical errors derived by our study design, we matched the samples, removing biases related to systemic factors influencing wound healing. Biases related to local influencing factors (such as previous treatments/surgeries performed) as well as operator-dependent biases were also removed by considering only previously untreated wounds (or treated just with basic wound care) and exclusively selecting procedures performed by the same surgeon.

Our study has some limitations. It was not randomized and this could represent a clear bias; patients are not randomized for the type of treatment but they were “enrolled” consecutively so, in our opinion, this is a little but valid expedient for bias reduction. We kindly encourage our colleagues to perform similar studies but trying to randomize the study cohort. Moreover, our sub-groups analysis sometimes led to very small samples but, as the reader can imagine, the “funnel effect” of a sub-groups analysis often led to a problem like this (despite the great number of initially enrolled cases) and for this reason, again, we kindly encourage our colleagues to perform other studies like this but trying to enlarge the study cohort.

## 5. Conclusions

This is the largest retrospective cohort, biases-reduced, multi-comparative study presented in literature among dermal substitutes in the treatment of critical soft-tissue wounds of the lower limb. Integra demonstrates the highest rate of skin graft viability and the highest rate of skin graft takes after 7 days. Pelnac^®^ shows the quickest induction of secondary healing in acute wounds as well as the best effectiveness in the overall supra-fascial (i.e., superficial) wounds closure. Nevelia^®^ is not different from Integra^®^ and shows a superior graft take compared to Pelnac^®^, but features the lowest secondary healing induction rate. No differences exist between the three DSs in terms of overall wound healing after 30 days from the skin graft or from the removal of the external silicon layer.

## Figures and Tables

**Table 1:** Patients’ characteristics as well as wounds’ features categorized according to the applied dermal substitute.

	Pelnac^®^ N (%)	Integra^®^ N (%)	Nevelia^®^ N (%)	Total
Cases	72	28	22	122
Comorbidities				
Diabetes	15 (21%)	8 (29%)	8 (36%)	*p* = ns
CVI	37 (51%)	15 (53%)	6 (27%)	*p* = ns
PVD	22 (30%)	8 (29%)	5 (23%)	*p* = ns
CAD	15 (21%)	12 (43%)	6 (27%)	*p* = ns
Other	23 (32%)	7 (25%)	8 (36%)	*p* = ns
Wound Depth				
Supra-fascial	67 (93%)	21 (75%)	13 (59%)	*p* = 0.001
Sub-fascial	5 (7%)	7 (25%)	9 (41%)	*p* = 0.001
Etiology				
Acute	21 (29%)	24 (86%)	13 (59%)	*p* < 0.001
Chronic	51 (71%)	4 (14%)	9 (41%)	*p* < 0.001
Microbiology				
Positive	46 (76%)	3 (11%)	8 (38%)	*p* < 0.001
Total	60	27	21	108
Skin Graft				
Total	17 (24%)	25 (89%)	15 (68%)	57, *p* < 0.001
Graft take				
Total	53%	92%	80%	*p* = 0.01
Micro (-)	57%	91%	82%	*p* = ns
Acute	63%	91%	75%	*p* = ns
% Wound healed	71%	63%	63%	*p* = ns

## Data Availability

The clinical data used to support the findings of this study are included within the article.
